# Calcium Ions Regulate K^+^ Uptake into Brain Mitochondria: The Evidence for a Novel Potassium Channel

**DOI:** 10.3390/ijms10031104

**Published:** 2009-03-12

**Authors:** Jolanta Skalska, Piotr Bednarczyk, Marta Piwońska, Bogusz Kulawiak, Grzegorz Wilczynski, Krzysztof Dołowy, Alexei P. Kudin, Wolfram S. Kunz, Adam Szewczyk

**Affiliations:** 1 Laboratory of Intracellular Ion Channels, Nencki Institute of Experimental Biology, 3 Pasteur st., 02–093 Warsaw, Poland; E-Mails: Jolanta_Skalska@URMC.Rochester.edu (J.S.); p.bednarczyk@nencki.gov.pl (P.B.); m.glab@nencki.gov.pl (M.P.); b.kulawiak@nencki.gov.pl (B.K.); adam@nencki.gov.pl (A.S.); 2 Department of Biophysics, Agricultural University SGGW, 159 Nowoursynowska St., 02–776 Warsaw, Poland; E-Mail: krzysztof_dolowy@sggw.pl; 3 Laboratory of Molecular and Systemic Neuromorphology, Nencki Institute of Experimental Biology, 3 Pasteur st., 02–093 Warsaw, Poland; E-Mail: g.wilczynski@nencki.gov.pl; 4 Division of Neurochemistry, Department of Epileptology and Life&Brain Center, University Bonn Medical Center, Sigmund-Freud-Str. 25, D-53105 Bonn, Germany; E-Mail: alexei.kudin@ukb.uni-bonn.de

**Keywords:** Mitochondria, brain, channel openers, potassium channel, iberiotoxin, NS1619

## Abstract

The mitochondrial response to changes of cytosolic calcium concentration has a strong impact on neuronal cell metabolism and viability. We observed that Ca^2+^ additions to isolated rat brain mitochondria induced in potassium ion containing media a mitochondrial membrane potential depolarization and an accompanying increase of mitochondrial respiration. These Ca^2+^ effects can be blocked by iberiotoxin and charybdotoxin, well known inhibitors of large conductance potassium channel (BK_Ca_ channel). Furthermore, NS1619 – a BK_Ca_ channel opener – induced potassium ion–specific effects on brain mitochondria similar to those induced by Ca^2+^. These findings suggest the presence of a calcium-activated, large conductance potassium channel (sensitive to charybdotoxin and NS1619), which was confirmed by reconstitution of the mitochondrial inner membrane into planar lipid bilayers. The conductance of the reconstituted channel was 265 pS under gradient (50/450 mM KCl) conditions. Its reversal potential was equal to 50 mV, which proved that the examined channel was cation-selective. We also observed immunoreactivity of anti-β_4_ subunit (of the BK_Ca_ channel) antibodies with ~26 kDa proteins of rat brain mitochondria. Immunohistochemical analysis confirmed the predominant occurrence of β_4_ subunit in neuronal mitochondria. We hypothesize that the mitochondrial BK_Ca_ channel represents a calcium sensor, which can contribute to neuronal signal transduction and survival.

## 1. Introduction

Mitochondria play a fundamental role in cerebral survival/death signaling pathways [9]. Moreover, under physiological and pathological conditions mitochondria actively direct the spatiotemporal calcium ion homeostasis within the cell. Calcium increases in the cytosol of neuronal cells due to synaptic transmission, excitoxicity or ischemic insults. Mitochondrial Ca^2+^ uptake is one of the most efficient ways of lowering calcium ions in neurons under physiological conditions [7]. The main function of mitochondrial Ca^2+^ appears to be the stimulation of oxidative phosphorylation [6]. On the other hand mitochondrial Ca^2+^ overload, which occurs during some pathological conditions, can lead to neuronal injury [28]. Calcium ions can trigger opening of permeability transition pores (PTP) in inner mitochondrial membrane, whose activation is associated with apoptosis via a mitochondrial pathway or necrosis due to mitochondrial damage [35]. Moreover, PTP opening by reactive oxygen species (ROS) is also enhanced by Ca^2+^; therefore, under the pathological condition of oxidative stress, impaired calcium ion homeostasis can lead to mitochondrial dysfunction [6].

Because Ca^2+^ ion homeostasis is crucial for cell physiology, the aim of the present study was to identify potential calcium ion-activated potassium transporting pathways in brain mitochondria. These could directly contribute to cellular ion signaling by modulation of mitochondrial membrane potential or the production of ROS and by affecting the activity of permeability transition pore. In this context it has been suggested that the integrity of mitochondrial inner membrane can be a target for new neuroprotective strategies [8].

We observed, with the use of isolated rat brain mitochondria, that Ca^2+^ ions cause an increase of respiratory rate of mitochondria in potassium medium, which was blocked by iberiotoxin (IbTx) and charybdotoxin (ChTx) – well known inhibitors of the large conductance potassium channel. The mitochondrial membrane potential was also decreased by calcium ions in the presence of K^+^, which was inhibited by IbTx and ChTx. Additionally, potassium channel openers, like NS1619, were able to modulate mitochondrial properties in a potassium selective way. Reconstitution of the inner mitochondrial membrane into planar lipid bilayers revealed the presence of a large conductance, calcium activated potassium (BK-type) channel in rat brain mitochondria. Finally, immunochemical studies suggested the presence of the BK-type channel β4 subunit in neuronal mitochondria. Thus, we propose the presence of a large conductance, calcium activated potassium channel in the inner membrane of brain mitochondria.

## 2. Results

### 2.1. Ca^2+^ induces BK channel inhibitor-sensitive potassium transport in isolated brain mitochondria

**Figure 1.**
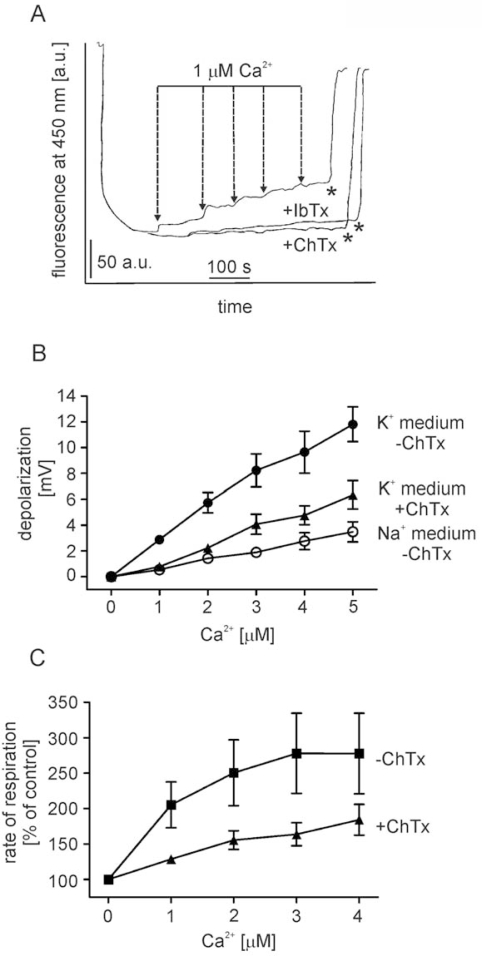
Effect of Ca^2+^ on the membrane potential of isolated brain mitochondria. (A) Original traces of mitochondrial membrane depolarization induced by Ca^2+^ in potassium medium (control), potassium medium supplemented with 50 nM IbTx and 200 nM ChTx. The mitochondrial membrane potential was measured by rhodamine 123 fluorescence at the concentration of 2.5 μM (as described in the Experimental section). The suspension of rat brain mitochondria was added into 1 mL of potassium medium with or without BK channel blockers; as respiratory substrates 5 mM malate and 10 mM glutamate were used. Complete membrane depolarization was reached by addition of 1.5 μM TTFB (indicated by *). (B) Quantitative changes of mitochondrial membrane potential after subsequent additions of calcium ions to mitochondria in potassium medium (filled circles), potassium medium supplemented with 200 nM charibdotoxin (filled triangles) and sodium medium (open circles). The experimental points are averages ±S.E.M of three independent experiments. The mitochondrial membrane potential was measured as described in the Experimental section. (C) Percentage of state 2 respiration stimulation of mitochondria by subsequent additions of CaCl_2_ into potassium containing medium, in the absence of BK channel inhibitors (filled squares) or in the presence of 200 nM ChTx (filled triangles). The mitochondrial respiration was measured as described in the Experimental section. The experiments are averages ±S.E.M of three independent experiments.

To investigate the possible effect of Ca^2+^ on potassium permeability of isolated rat brain mitochondria we determined the mitochondrial membrane potential in sodium- and potassium-containing media in the presence of different Ca^2+^ concentrations. Measurements of mitochondrial membrane potential were performed with the use of the potential sensitive fluorescence dye rhodamine 123. First, we measured the mitochondrial membrane potential in potassium medium (Figure 1A). Additions of Ca^2+^ up to the concentration of 5 μM caused mitochondrial depolarization visible as pronounced increased in rhodamine 123 fluorescence (Figure 1A, upper trace). To exclude the potential involvement of electrophoretic Ca^2+^ entry as a cause of the observed mitochondrial membrane dissipation at applied Ca^2+^ concentrations, we tested the mitochondrial membrane depolarization evoked by Ca^2+^ in sodium medium. The obtained results are illustrated on Figure 1B (open circles). Ca^2+^ cycling and calcium/sodium exchange is unlikely to contribute to the observed effect, since the depolarization evoked by 5 μM Ca^2+^ in sodium medium is lower (∼ 3 mV, Figure 1B, open circles) in comparison to potassium medium (∼ 13 mV, Figure 1B, filled circles). Moreover, the Ca^2+^ -induced, potassium – specific depolarization is sensitive to 50 nM IbTx (Figure 1A, middle trace) or 200 nM ChTx (Figure 1A, lower trace, and Figure 1B, filled triangles). We also observed that Ca^2+^ induced an increase in respiration of brain mitochondria in potassium medium (Figure 1C, filled squares), which was sensitive to 200 nM ChTx (Figure 1C, filled triangles). These results are in line with the data obtained from the potential measurements, as an increase of potassium flux into mitochondrial matrix is usually related to an increase in oxygen consumption.

**Figure 2.**
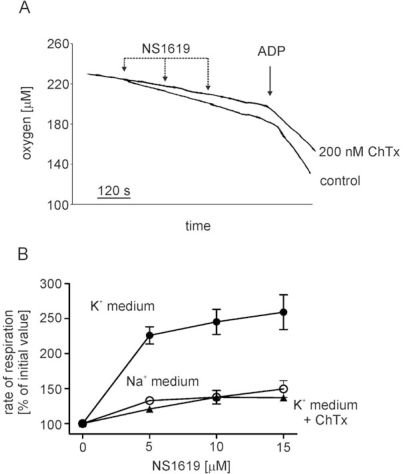
Potassium channel opener NS1619 induces charybdotoxin sensitive increase of rat brain mitochondria respiration. (A) Original oxygraph trace illustrating the effect of large conductance potassium channel opener NS1619 on the rate of respiration of mitochondria isolated from rat brain. Additions: NS1619 – 5 μM, ChTx – 200 nM, ADP – 100 μM. (B) Percentage of stimulation of mitochondrial state 2 respiration by subsequent additions of NS1619 in potassium – containing medium (filled circles), sodium – containing medium (open circles) and potassium –containing medium supplemented with 200 nM ChTx (filled triangles). The experimental points are average ±S.E.M of three independent experiments.

These observations suggested the presence of a large conductance Ca^2+^ activated potassium channel in inner mitochondrial membrane of rat brain mitochondria. Therefore we tested the effect of the BKC_a_-channel opener NS1619 on mitochondrial respiration. As presented in Figures 2A and 2B (filled circles) the addition of NS1619 at the concentration up to 15 μM caused a ChTx – sensitive increase in respiration (Figures 2A and 2B, filled triangles). The NS1619 additions were without effect on mitochondrial respiration in sodium medium (Figure 2B, open circles).

### 2.2. Reconstitution of inner mitochondrial membranes into planar lipid bilayers

**Figure 3.**
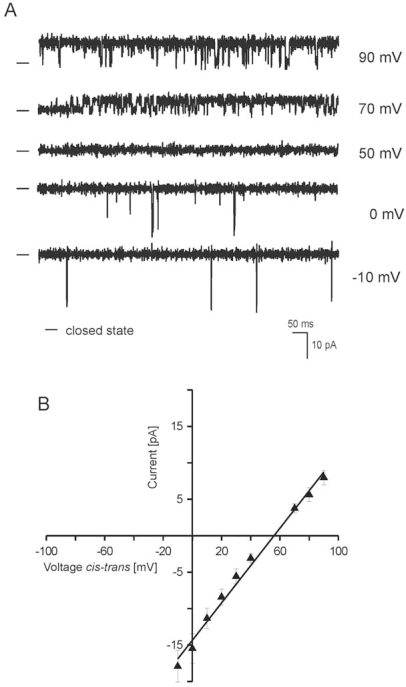
Single-channel recordings of the rat brain mitoBK_Ca_ channel in planar lipid bilayers. (A) Single-channel recordings in gradient 50/450 mM KCl (*cis/trans*) solution at different voltages. – indicates the closed state of the channel. Recordings were low pass filtered at 1 kHz; (B) Current-voltage characteristics of single-channel events in gradient 50/450 mM KCl.

**Figure 4.**
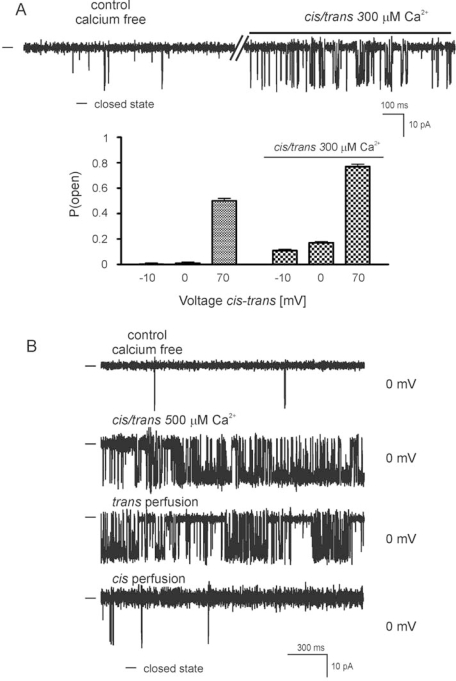
Ca^2+^ effects on the activity of the mitoBK_Ca_ channel. (A) Single channel recordings in gradient 50/450 mM KCl (*cis/trans*) solution at 0 mV under calcium free control conditions and after addition of 300 μM Ca^2+^ (*cis/trans*). – indicates the closed state of the channel. Recordings were low pass filtered at 1 kHz. Lower panel: P(open) value at different voltages under calcium free control conditions and after addition of 300 μM Ca^2+^ (*cis/trans*). (B) Single channel recordings in gradient 50/450 mM KCl (*cis/trans*) solution at 0 mV under calcium free control conditions, after addition of 500 μM Ca^2+^ *(cis/trans),* and after perfusion: first *trans* side and next *cis* side. – indicates the closed state of the channel. All recordings were low pass filtered at 1 kHz.

Preparations of inner mitochondrial membrane particles (SMP) from rat brain were reconstituted into black lipid membranes and the current characteristics of ion channels were observed. The fusion of SMP to the bilayer was usually observed within 15–30 min after addition to the *trans* compartment. Original single channel recordings in gradient 50/450 mM KCl (*cis/trans*) solution at different voltages are shown in Figure 3A. Figure 3B shows the current-voltage relationships for single channel opening at different voltages under gradient conditions. The channel conductance was 265 ± 5 pS under gradient conditions. The reversal potential measured in the gradient 50/450 mM KCl (*cis/trans*) solution is equal to 50 mV and this proves that the examined channel is cation-selective (Figure 4A). The mean reversal potential calculated after fitting to experimental data is equal to 52 ± 4 mV. The distribution of open and closed dwell-time was also analyzed. The open dwell-times at 90 mV and −10 mV is equal to approximately 10 ms and 1 ms, respectively; as well as closed dwell-times at 90 mV and −10 mV are equal to approximately 1 ms and 602 ms, respectively. This means that the channel at gradient conditions over reversal potential was mainly open and at negative holding potential was practically closed.

Substances known to modulate the mitoBK_Ca_ channel activity were also used to examine the ion channel properties. Figure 4A shows single channel recordings in gradient 50/450 mM KCl (*cis/trans*) solution at 0 mV under calcium free control condition and after addition of 300 μM Ca^2+^ (*cis/trans*). We observed an increase probability of channel opening in presence of 300 μM Ca^2+^ *(cis/trans):* e.g. P(open) at 70 mV increase from 0.50 ± 0.02 to 0.77 ± 0.02. Figure 4B shows single channel recordings in gradient 50/450 mM KCl(*cis/trans*) solution at 0 mV under calcium nominally free control condition, after addition of 500 μM Ca^2+^ (*cis/trans*) and after perfusion first *trans* and second *cis* compartment. Changes of channel activity were observed after addition 500 μM Ca^2+^ *(cis/trans).* Perfusion of the *trans* compartment were without effect on the mitoBK_Ca_ channel activity but after perfusion of the *cis* compartment a return of channel activity to the control activity was observed.

**Figure 5.**
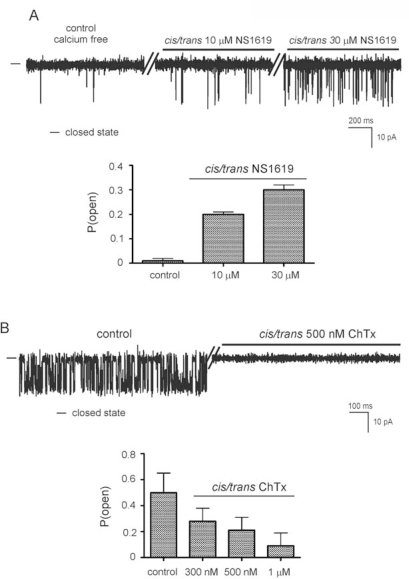
NS1619 and charybdotoxin modulate the activity of the mitoBK_Ca_ channel. (A) Single channel recordings in gradient 50/450 mM KCl (*cis/trans*) solution at 0 mV under calcium free control conditions, after addition of 10 μM NS1619 *(cis/trans),* and after addition of 30 μM NS1619 *(cis/trans).*– indicates the closed state of the channel. Recordings were low pass filtered at 1 kHz. Lower panel: P(open) value under calcium free control conditions and at 10 and 30 μM NS1619 (*cis/trans*). (B)Single channel recordings in gradient 50/450 mM KCl (*cis/trans*) solution at 0 mV under control conditions and after addition of 500 nM ChTx *(cis/trans).* – indicates the closed state of the channel. Recordings were low pass filtered at 1 kHz. Lower panel: P(open) value under control conditions and at different concentration ChTx *(cis/trans).* Control measurements were performed in the presence of 1 mM Ca^2+^ *(cis/trans).*

The potassium channel opener NS1619 known to modulate the mitoBK_Ca_ channel activity was used to alter the ion channel properties observed in our experiments. Figure 5A shows single channel recordings in gradient 50/450 mM KCl (*cis/trans*) solution at 0 mV under calcium nominally free control condition and after addition of 10 μM or 30 μM NS1619 *(cis/trans).* The probability of opening of the ion channel increased from 0.01 ± 0.01 to 0.30 ± 0.02 in presence of 30 μM NS1619 in *cis/trans* compartments.

The effects of ChTx on mitoBK_Ca_ channel activity were examined in detail. The single channel recordings in gradient 50/450 mM KCl (*cis/trans*) solution at 0 mV under control condition and after addition 500 nM ChTx *(cis/trans),* which inhibits channel activity, are presented in Figure 5B. Control measurements were performed in the presence of 1 mM Ca^2+^ *(cis/trans).* The dose-response effect of probability of opening mitoBK_Ca_ channel after addition 300 nM, 500 nM and 1 μM ChTx *(cis/rans)* was determined. The P (open) of mitoBK_Ca_ channel decreased from 0.50 ± 0.10 at control conditions to 0.09 ± 0.05 in the presence of 1 μm ChTx in *cis/trans* compartments.

### 2.3. Identification of a BK channel subunit in brain mitochondria

**Figure 6.**
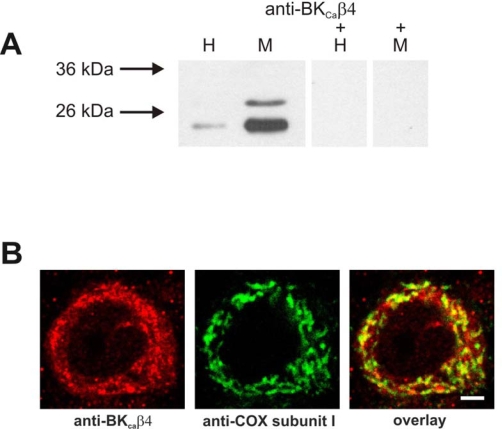
Immunodetection of BK_Ca_ β_4_ subunit in rat brain mitochondria. (A) Western blot detection of BK_Ca_ β4 subunit in rat brain mitochondria. Immunoblot analysis of homogenate (H) and mitochondrial fraction (M) of rat brain revealed the presence of BK_Ca_β4 – immunoreactive product of approximately 26 kDa in mitochondria. The immunoreaction was displaced with an antigen peptide (M+). (B) Mitochondrial localization of BK_Ca_ β_4_ subunit in rat hippocampal neurons. High power confocal image of neuron in rat hippocampal CA1 region immunolabeled for the BKCa β_4_ subunit (red) and the mitochondrial marker COX subunit I (green). The yellow color in the overlay indicates colocalization. Scale bar = 3 μm.

In order to localize the mitochondrial BK_Ca_ channel protein immunochemical techniques were applied. The potential presence of BK_Ca_ channel subunits in purified brain mitochondria was analyzed by *Western blotting* (Figure 6A). The purity of isolated brain mitochondria was confirmed with the use of antibody against ATP/ADP translocase (ANT), a marker enzyme of inner mitochondrial membrane. The results (with the use of antibodies against plasma membrane BK_Ca_ channel subunits) suggest the presence of the β_4_ subunit of BK_Ca_ with an apparent molecular weight ~26 kDa in the mitochondrial fraction. A specific immunostaining with anti-BK_Ca_ subunit α was however not observed with the applied antibody (data not shown, see Experimental section).

To determine the distribution of BK_Ca_ channel in rat brain tissue we applied immunohistochemical studies with the use of antibodies against BK_Ca_ α subunit (data not shown) and β_4_ subunit. No specific anti-BK_Ca_ α subunit immunoreactivity was observed, whereas the distribution pattern of β_4_ subunit revealed a preferentially neuronal localization. To investigate further the subcellular localization of BK_Ca_ channel in brain, we co-immunostained the sections with anti-BK_Ca_ β_4_ subunit antibody and cytochrome c oxidase antibody as a mitochondrial marker (Figure 6B). As presented in Figure 6B, both antibodies yielded similar patterns of immunoreactivity showing a significant colocalization. However, not the whole amount of BK_Ca_ β_4_ immunofluorescence signal was found to be co-localized with mitochondria. As visible in the overlay, also a minor fraction of the mitochondrial staining appeared to be not co-localized with anti-BK_Ca_ β_4_ subunit antibody signal.

## 3. Discussion

Ion channels selective for potassium ions are present in the inner mitochondrial membrane [15,25,29]. These include the ATP-regulated potassium channel (mitoK_ATP_ channel), the large conductance, calcium activated potassium channel (mitoBK_Ca_ channel) and a voltage dependent potassium channel (mitoKv1.3 channel) (for reviews see [5,17,30,32]). The mitoK_ATP_ channel attracts attention due to its likely involvement in cytoprotective phenomena in cardiac [33] and in brain [20,21] tissues. The mitoK_ATP_ channel was found in skeletal muscle mitochondria [12] and human T-lymphocytes [10]. Additionally, a neuroprotective role of the mitoK_ATP_ channel present in brain mitochondria was postulated [8].

A putative mitochondrial large conductance Ca^2+^-activated potassium channel (mitoBK_Ca_ channel) was originally described using patch-clamp technique in human glioma cells LN229 [25]. This channel, with a conductance of 295 pS, was stimulated by Ca^2+^ and blocked by charybdotoxin. Later the presence of a channel with properties similar to the plasma membrane calcium-activated K^+^ channel (stimulated by the potassium channel opener NS1619 and blocked by ChTx, IbTx and paxilline) was observed in patch-clamp recordings from mitoplasts of guinea pig ventricular cells [34]. Immunoblots of cardiac mitochondria with antibodies against the C terminal part of BK_Ca_ channel identified a 55 kD protein as putative channel which may contribute to the cardioprotective effect of K^+^ influx into mitochondria. Additionally, it has been reported that the activation of the cardiac mitoBK_Ca_ by NS1619 (measured by flavoproteins oxidation) is amplified by 8-bromoadenosine-3’,5’-cyclic monophosphate and forskolin [24]. This suggests that the opening of mitoBK_Ca_ is modulated by a cAMP-dependent protein kinase.

It should be mentioned, that the mitoBK_Ca_ channel may offer a novel link between cellular/mitochondrial calcium signaling and mitochondrial membrane potential-dependent reactions. Altered intramitochondrial calcium levels directly affect the potassium permeability of the mitochondrial inner membrane thus modulating the membrane potential. This type of interaction can modulate the efficiency of oxidative phosphorylation in a calcium-dependent manner. Very likely, the mitochondrial channel has its charybdotoxin/iberiotoxin binding site close to the cytosolic compartment since those compounds as peptides cannot easily enter the mitochondrial matrix space. Consequently, the calcium binding site of the mitoBK_Ca_ channel is close to the matrix compartment. Recently, with the use of immunochemistry and immuno-gold electron microscopy it was suggested that a mitoBK_Ca_ channel might be present also in brain mitochondria [23]. Hence, in the present study we were searching for functional evidence of presence of a mitoBK_Ca_ channel in brain mitochondria.

Our studies, performed with isolated mitochondria and with the planar lipid bilayer technique confirm the functional presence of a mitoBK_Ca_ channel in brain mitochondria. This channel has the following properties:

- the channel (selective for potassium) is activated by calcium ions (probably from the matrix side) and is sensitive to ChTx and IbTx,
- the channel is activated by the potassium channel opener NS1619,
- the channel has a conductance of 265 pS and is voltage dependent.

Furthermore, with the use of immunochemical techniques we have shown the presence of β_4_ subunit of the BK_Ca_ channel in brain mitochondria. Probably, the channel is predominantly present in mitochondrial of neuronal cells. Recently, the detailed distribution pattern of the β4 subunit of BK_Ca_ channel in brain tissue was published [23].

Similar to the mitochondrial ATP-regulated potassium channel, this novel potassium channel is expected to affect mitochondrial metabolism due to regulation of matrix volume. In fact, we have shown that mitoBKCa channel activation in brain mitochondria can modulate mitochondrial respiration and mitochondrial membrane potential. Recently, a functional active mitoBK_Ca_ channel was also identified in skeletal muscle mitochondria [26].

In addition to classical physiological effects of mitochondrial potassium transport, a pivotal role of mitochondrial potassium channels has been implicated for neuroprotection. It is therefore reasonable to expect a possible neuroprotective effect of mitoBK_Ca_ channel activation, probably due to the modulation of synthesis of reactive oxygen species [19]. In order to finally clarify the potential neuroprotective role of the mitoBK_Ca_ channel a clear distinction has to be done between the plasma membrane and the mitochondrial BK_Ca_ channel. This issue is complicated by the similar pharmacological properties of mitochondrial and plasma membrane BK_Ca_ channels, both at the level of channel blockers and potassium channel openers. Further complications arise from the relatively low specificity of BK_Ca_ channel openers, like NS1619 (cf [11,18]), which have to be overcome by careful control experiments with the use of the specific blockers ChTx and IbTx, acting in the nmolar concentration range.

## 4. Experimental Section

### 4.1. Materials

L-α-Phosphatidylcholine (asolectin) and *n*-decane were from Sigma-Aldrich, Germany. All other chemicals were of the highest purity available commercially.

### 4.2. Isolation of rat brain mitochondria and submitochondrial particles

Rat brain mitochondria were prepared according to a standard protocol. Shortly, a male *Wistar* rat was anesthesied with chloroform and then euthanized by cervical dislocation. The brain was rapidly removed, washed and placed in ice-cold buffer containing 225 mM mannitol, 75 mM sucrose, 5 mM HEPES, BSA (1 mg/mL), 1 mM EGTA, pH 7.4. The tissue was then minced with scissors and placed in 10 mL of isolation medium supplemented with nagarase (0.5 mg/mL) and then homogenized with the use of a motor-driven Teflon-glass Potter homogenizer. After two-fold dilution, the homogenate was centrifuged at 2,000 × g for 9 min. The supernatant was decanted and centrifuged at 12,000 × g for 11 min. To permeabilize synaptosomes, the pellet was suspended in isolation buffer supplemented with digitonin (0.2 mg/mL) and homogenized manually. Finally the suspension was centrifuged at 12,000 × g for 11 min. The mitochondrial pellet was resuspended in isolating medium at a protein concentration of 20–40 mg/mL. All procedures were carried out at 4°C. To obtain submitochondrial particles (SMP) the rat brain mitochondria were thawed, sonicated 3 × 15 s and ultracentrifuged. The submitochondrial particles were resuspended at a final concentration of about 4 mg protein/mL.

### 4.3. Mitochondrial membrane potential measurements

The measurements were made at room temperature in the 1 mL cuvette of a Shimadzu RF-5001 spectrofluorometer (Tokyo, Japan) using 2.5 μM rhodamine 123, a membrane potential sensitive fluorescence dye. The measurements were performed in a medium containing 10 mM KH_2_PO_4_ (or NaH_2_PO_4_), 60 mM KCl (or NaCl), 60 mM Tris, 5 mM MgCl_2_, 110 mannitol and 0.5 mM EDTA-Na_2_, pH 7.4. For the measurements of calcium effects on mitochondrial potential, the medium contained 10 mM KH_2_PO_4_ (or NaH_2_PO_4_), 60 mM KCl (or NaCl), 60 mM Tris, 5 mM MgCl_2_, 110 mannitol and 5 μM EDTA-Na_2_, pH 7.4. As respiratory substrates 10 mM glutamate and 5 mM malate were used. The protein concentration of rat brain mitochondria was 0.4 – 0.5 mg protein/mL. The samples were excited at 450 nm and the fluorescence was registered at 550 nm. To calculate the mitochondrial membrane potential, the fluorescence changes were calibrated using corresponding potassium diffusion potentials. For this, 2 μg/mL valinomycin was added to the rotenone de-energized mitochondrial preparation, incubated in a medium in which K^+^ was replaced by Na^+^. Thereafter, defined additions of KCl were made until no changes of rhodamine 123 fluorescence were recorded (equilibrium point). The corresponding potential was calculated with the Nernst equation.

### 4.4. Mitochondrial respiration

Mitochondrial oxygen consumption was measured at 25°C using an Oroboros oxygraph (Anton Paar, Austria) in a medium containing 10 mM KH_2_PO_4_ (or NaH_2_PO_4_), 60 mM KCl (or NaCl), 60 mM Tris, 5 mM MgCl_2_, 110 mannitol and 0.5 mM EDTA-Na_2_, pH 7.4 (or with 5 μM EGTA for measurements in the presence of calcium chloride) and 10 mM glutamate and 5 mm malate as respiratory substrates. The concentration of mitochondria was 0.3 mg protein/mL.

### 4.5. Single channel measurements with black lipid membrane (BLM) technique

BLMs were formed in a 250 μm diameter hole drilled in a Delrin cup (Warner Instrument Corp., Hamden, CT USA), which separated two chambers (*cis* and *trans* 1 mL internal volume). The chambers contained 50/450 mM KCl *(cis/trans),* 20 mM Tris-HCl, pH 7.2 solutions. The outline of the aperture was coated with a lipid solution and N_2_ dried prior to bilayer formation to improve membrane stability. BLMs were painted using asolectin in *n*-decane at a final concentration of 25 mg lipid/mL. Rat brain SMP (4 mg of protein/mL, 1–3 μL) were added to the *trans* compartment. Incorporation of the potassium channel into the BLM was usually observed within few minutes. The studied compounds were added to *cis* or *trans* compartments. All measurements were carried out at air conditioned room temperature (24°C). Formation and thinning of the bilayer was monitored by capacitance measurements and optical observations. Final accepted capacitance values ranged from 110 to 180 pF. Electrical connections were made by Ag/AgCl electrodes and agar salt bridges (3 M KCl) to minimize liquid junction potentials. Voltage was applied to the *cis* compartment of the chamber and the *trans* compartment was grounded. The current was measured using a Bilayer Membrane Amplifier (BLM-120, BioLogic). For removing solutions from *cis* or *trans* compartments we used a perfusion system. This system consisted of a holder with glass tip, a perfusion pump (sp260p syringe pump, USA), a glass syringe (FORTUNA OPTIMA Glasspritze, Aldrich) and Teflon tubing (C-FLEX TUBING, Sigma).

### 4.6. Data analysis

Signals were filtered at 1 kHz. The current were digitized at a sampling rate of 100 kHz (A/D converter PowerLab 2/25, ADInstruments) and transferred to a PC or digital tape recorder (DTR-1204, BioLogic) for off-line analysis by Chart v5.2 (PowerLab ADInstruments) and pCLAMP8.1 (Axon Laboratory). The pCLAMP8.1 software package was used for data processing. The channel recordings illustrated are representative of the most frequently observed conductances under the given conditions. The conductance was calculated from the current-voltage relationship. Single channel currents were recorded at different voltages: e.g. 90, 70, 50, 0, −10, −30 (in mV). The probability of a channel opening (P(open)) was calculated with automatic interval setting. The channel open (τ_open_) and closed (τ_closed_) lifetimes were calculated from logarithmic binning mode using Marquardt-LSQ fitting method, order one, without weighting. *n* denotes number of experiments, and N the number of events.τ_open_, τ_closed_, P(open) were calculated from segments of continuous recordings lasting 60 s and with N ≥ 2000 events. Data from experiments are reported as mean value or value ± SD (standard deviation).

### 4.7. Western blot analysis

For immunoblot staining, the proteins from brain mitochondria were precipitated with the use of 20 % ice-cold trichloroacetic acid. After 30 min incubation on ice samples were centrifuged at 15,000 × g at 4°C for 15 min. The pellet was then suspended in Laemmli loading dye supplemented with triethanoloamine and boiled for 10 min. Equal amounts of 30 μg of protein was resolved on a 12 % SDS-PAGE, and electroblotted onto a nitrocellulose membrane (BIO-RAD). The membranes were blocked for 2 hours at room temperature with 10% non-fat milk in TBS-T buffer (20 mmol/l Tris, 137 mmol/l NaCl, 0.1 % Tween 20, pH 7.6) and incubated overnight with specific primary antibody (anti-BK_Ca_ subunit β4, N-terminal specific and α, C-terminus specific, Alamone Lab). After washing, the blots were incubated for 1 hour with a horseradish peroxidase-conjugated secondary antibody (Amersham Pharmacia Biotech, UK) and were viewed using an ECL detection system (Amersham PharmaciaBiotech, UK).

### 4.8. Immunochemistry

Adult *Wistar* rats were perfused with 4% paraformaldehyde in accordance with the rules established by the Ethical Committee on Animal Research of the Nencki Institute. The brains were removed, immersed in 4% paraformaldehyde for 24 h, washed in phosphate buffer saline (PBS) and paraffin embedded. Coronal sections (5 μm thick) were cut and mounted onto silane-coated slides. For antigen retrieval, the dewaxed and rehydrated sections were heated in microwave oven in citrate buffer (pH 6.0) for 2x7 min, followed by 20 min cooling in the same buffer.

Nonspecific antibody binding sites were blocked by preincubating sections with 5% normal donkey serum (NDS) diluted in PBS for 30 min. For the simultaneous detection of the BK_Ca_β_4_ subunit with the neuronal marker MAP2 (microtubule associated protein 2) or the mitochondrial marker cytochrome c oxidase, sections were incubated overnight at 4°C in 5% NDS containing a mixture of primary antibodies: rabbit polyclonal anti-BK_Ca_ β_4_ subunit (KCNMB4) antibody (1:50 dilution, Alomone Labs) and mouse monoclonal anti-MAP2 antibody (1:100 dilution, Chemicon) or mouse monoclonal anti-OxPhos Complex IV subunit I (1:200 dilution, Molecular Probes). The immunoreaction was visualized using Alexa-Fluor 488 conjugated donkey anti-mouse antibody and Alexa-Fluor 555 conjugated donkey anti-rabbit antibody (Molecular Probes). The sections were then mounted in VECTASHIELD HardSet Mounting Medium with DAPI (Vector). Confocal images were acquired using a Leica microscope, model TCS SP2 using Ar laser (488 nm) and GeNe (543 nm) laser lines for the excitation of Alexa-Fluor 488 and Alexa-Fluor 555 respectively. To avoid cross talk between two fluorophores special care was taken to adjust detector settings, and the images were scanned sequentially.

## 5. Conclusions

Our studies, performed with the isolated rat brain mitochondria and with the planar lipid bilayer technique confirm the presence of a functional mitoBK_Ca_ channel in brain mitochondria. Immunochemical analysis confirmed the predominant occurrence of β_4_ subunit in neuronal mitochondria. We hypothesize that the mitoBK_Ca_ channel represents a new intracellular calcium sensor, which could potentially contribute to neuronal signal transduction. In addition, we propose that the activation of this potassium channel would lead in brain tissue to a neuroprotective effect, which is realized by inhibition of synthesis of reactive oxygen species [19].

## Abbreviations:

ANT: mitochondrial adenine nucleotide translocase;
BK_Ca_: channel, large conductance Ca^2+^-activated potassium channel;
BLM: black lipid membrane;
ChTx: charybdotoxin; IbTx, iberiotoxin;
NS1619: 1,3-dihydro-1-[2-hydroxy-5-(trifluoro-methyl)phenyl]-5-(trifluoromethyl)-2H-benzimidazole-2-one;
mitoBK_Ca_: channel, mitochondrial large conductance Ca^2+^ - activated potassium channel;
mitoK_ATP_: channel, mitochondrial ATP-regulated potassium channel;
ROS: reactive oxygen species;
SMP: submitochondrial particles;
EGTA: ethylene glycol–bis (β-amino–ethylether)–N,N,N’,N’, -tetraacetic acid;
TTFB: 4,5,6,7-tetrachloro-2-trifluoromethyl-benzimidazole.
